# Autonomic Self-Healing of Polymers: Mechanisms, Applications, and Challenges

**DOI:** 10.3390/molecules30030469

**Published:** 2025-01-22

**Authors:** Chenxu Wang, Roman Boulatov

**Affiliations:** 1 College of Chemistry and Chemical Engineering, Xi’an University of Science and Technology, Xi’an 710054, China; chenxuwang@xust.edu.cn; 2 Department of Chemistry, University of Liverpool, Crown Street, Liverpool L69 7ZD, UK

**Keywords:** polymer mechanochemistry, self-healing, mechanochemical degradation

## Abstract

Mechanical loads degrade polymers by enabling mechanochemical fragmentation of macromolecular backbones. In most polymers, this fragmentation is irreversible, and its accumulation leads to the appearance and propagation of cracks and, ultimately, fracture of the material. Self-healing describes a diverse and loosely defined collection of approaches that aim at reversing this damage. Most reported synthetic self-healing polymers are non-autonomic, i.e., they require the user to input free energy (in the form of heat, irradiation, or reagents) into the damaged material to initiate its repair. Here, we critically discuss emerging chemical approaches to autonomic self-healing that rely on regenerating the density of load-bearing, dissociatively-inert backbone bonds either after the load on a partially damaged material dissipated or continuously and in competition with the mechanochemically driven loss of backbones in the loaded material. We group the reported chemistries into three broad types whose analysis yields a set of criteria against which the potential of a prospective approach to yield practically relevant self-healing polymers can be assessed quantitatively. Our analysis suggests that the direct chain-to-chain addition in mechanically loaded unsaturated polyolefins is the most promising chemical strategy reported to date to achieve autonomic synchronous self-healing of practical significance.

## 1. Introduction and Background Concepts

Self-healing materials are designed to mimic the ability of living organisms to recover their shape and functions after suffering physical damage. In theory, self-healing confers multiple advantages, including improved reliability and durability, broadened applications, reduced maintenance costs, waste generation, and resource consumption [1]. Self-healing materials are thought to have the greatest potential impact in medical sciences, aerospace, electronics, automotive, and construction [2,3,4,5,6]. Self-healing polymeric materials are among the most extensively researched self-healing materials. The vast design space of polymeric systems allows considerable freedom in selecting chemistries, compositions, chain topologies, and microstructures to promote regeneration [7,8,9], potentially tuned to specific service conditions. The demonstrated approaches to self-healing vary greatly in terms of timescales, the requirements of free energy inputs (including supply of external reagents), and the extent to which the bulk mechanical properties are recovered.

At the molecular level, mechanical damage reduces the volumetric density of the backbone bonds resulting from mechanochemical fracture of polymer chains [10,11,12]. An above-a-threshold reduction in this density produces new interfaces, initially as microcracks and eventually as fractures of a whole sample [13,14]. Rebonding across such mechanically generated interfaces requires some bulk mass flows [7] and may be enhanced by the formation of new bonds if the delivered material is reactive. The requisite mass flow has been achieved by numerous strategies, e.g., chain interdiffusion (often driven by maximization of van der Waals [15], H-bonding [16], or other weak interactions instead of entropy alone [17]), shape-memory effects [18], localized melting, microvascularization, and encapsulation [19], and has been extensively reviewed. Reversible dissociation of covalent crosslinks at temperatures > 100 °C (e.g., Diels–Alder (DA) adducts) has been demonstrated to accelerate chain-interdiffusion and shape-memory-based rebonding by increasing chain mobility [20,21].

The capacity to rebond across mechanically generated interfaces, particularly at the macroscale, offers clearly exploitable opportunities [14,22]. A material capable of repairing mechanically generated sub-μm scale damage that precedes the microcracks formation has many additional advantages. Such molecular repairs potentially minimize the probability of a catastrophic material failure and increase its fatigue resistance and the duration the material can be used continuously without needing to be taken out of service.

**Figure 1:**
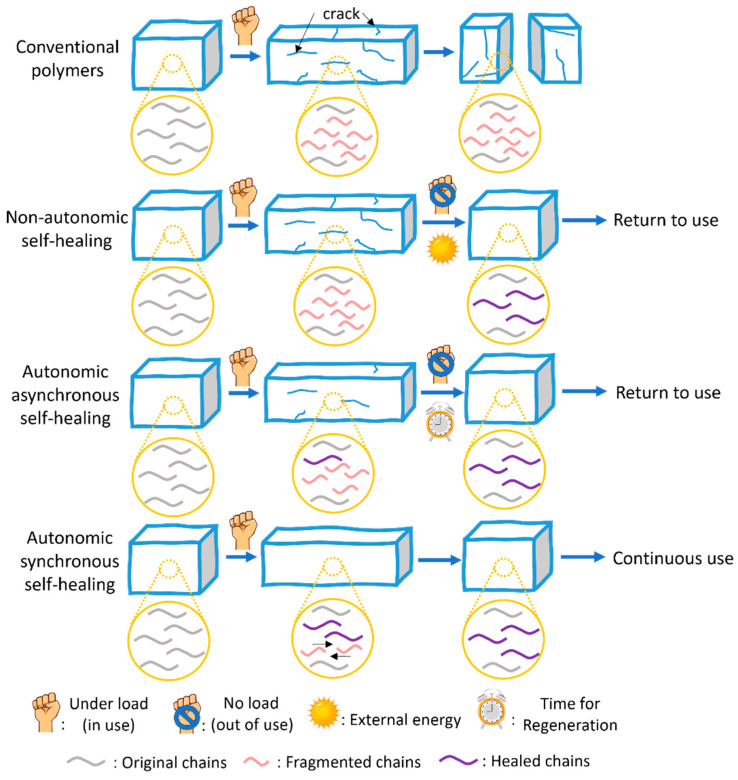
A cartoon representation of a conventional (non-self-healing) polymer and the 3 distinct types of self-healing polymers.

**Figure 2:**
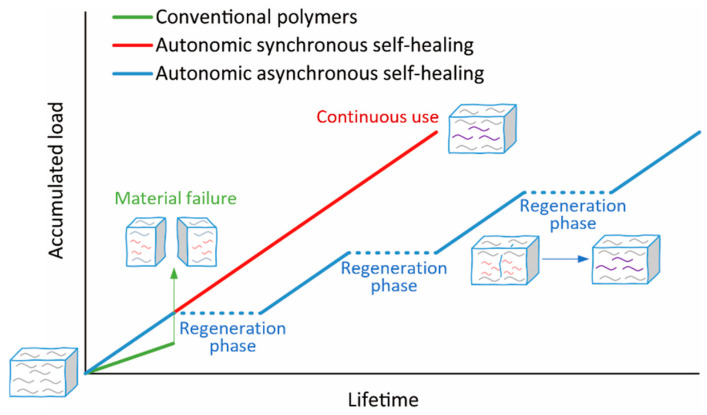
A cartoon representation of mechanical-load accumulation (which is used here as a proxy for useful lifetime) in polymers of different self-healing properties. The lines terminate when the material properties degrade below the acceptable threshold. Non-autonomic self-healing can resemble the trend for autonomic asynchronous self-healing (blue line). The plateaus in autonomic asynchronous self-healing (no change in the accumulated load) correspond to “regeneration” phases where the material is removed from use and allowed to recover.

We find it productive to distinguish three regimes of self-healing (Figure 1 and Figure 2):

(a) Non-autonomic self-healing requires free energy input (heat, irradiation, and/or the addition of a reagent) to initiate repair. Most reported self-healing polymers are non-autonomic because the exploitation of external energy input significantly expands the range of chemistries that are available for repair, can accelerate healing, and enhance the mechanical properties of the recovered materials. The most significant and inevitable drawback of such materials is that they cannot be healed in service and in situ, which limits their (prospective) applications.
(b) In autonomic asynchronous self-healing, the material (partially) regenerates its bulk mechanical properties spontaneously once the (mechanical) load dissipates without the need for external energy input. Most reported autonomic self-healing is asynchronous. Such materials can self-heal in situ if they are not subject to load during this regeneration phase, i.e., they usually must be removed from service to avoid unrepairable mechanical degradation. The “regeneration” phase, when the material cannot sustain useful mechanical load, is usually longer than the usable phase.
(c) In autonomic synchronous self-healing, the material regenerates its damaged molecular network simultaneously and in competition with the mechanochemical network degradation. These materials can be used continuously and do not require a separate “regeneration” phase or treatment. They are the rarest examples of self-healing at practically relevant loads and the ones with the highest technological or commercial potential. However, molecular-level autonomic synchronous self-healing imposes demanding constraints on the underlying chemistry (e.g., fast kinetics at low temperatures, no exogenous reagents), making it challenging to achieve.

Rebonding across macroscopic mechanically generated interfaces is necessarily non-autonomic because it requires the rebonding volumes to be brought into close contact by an external action (typically a compressive load), which is usually distinct from the mechanical load that causes material fracture (i.e., shear or tensile). Conversely, the molecular-level regeneration and rebonding across sub-μm-length interfaces is achievable, at least theoretically, in all three regimes.

Although rebonding across interfaces at μm- and longer scales is often confirmed visually, it is not a sufficient criterion of repair and is inapplicable at smaller length scales. Instead, a claim of self-healing requires a demonstration of (at least partial) regeneration of one or more bulk mechanical properties of the degraded material (regimes 1–2) or a demonstration that the material retains these properties under conditions where a suitable control degrades (regime 3). The bulk properties whose regeneration or maintenance under normally destructive loads were demonstrated include moduli, failure stress or strain, the critical energy release rate, onset of strain-hardening, energy dissipation capacity, and effective crosslinking density [23]. For non-covalently crosslinked polymers (i.e., melts and glasses), changes in the distribution of chain sizes and microstructures also reveal the regeneration of mechanically lost load-bearing bonds. However, no consensus quantitative definition of self-healing exists, and many claims in the literature on self-healing rely on qualitative observations and/or idiosyncratic definitions of self-healing. Little data exist in the literature to judge how robust diverse self-healing strategies are either to environmental factors (temperature, humidity, small-molecule pollutants) or repeated cycles of damage/repair. For non-autonomically self-healing polymers, the effect of the time lag between the material fracture and the healing intervention is also poorly studied, whereas the capacity of a self-healing material to rebond across mechanically generated macroscopic interfaces reduces rapidly the longer these interfaces are exposed to the environment.

An elastomer or a hydrogel fractures when it loses a sufficient number of backbone bonds for its molecular network to depercolate [10]. Most networks are chemically heterogeneous, i.e., they contain different chemical bonds. For simplicity, these bonds can be separated into dissociative labile and dissociatively inert types [24]. The most common dissociatively inert bond in synthetic polymers is C(sp^3^)-C(sp^3^). In the absence of a mechanical load, the probability of its spontaneous dissociation is negligible at temperatures up to at least 150 °C. This survival probability decreases monotonically with increasing load but remains negligible up to ~4.5 nN [25]. The importance of mechanochemical fracture of C backbones in the mechanical aging of commercial polymers [26] illustrates that backbone bonds at least occasionally experience higher forces. The resultant backbone fracture generates macroradicals [11]. Although these macroradicals have some probability of recombining to regenerate the lost bond, their often-high reactivity means that competing reactions (e.g., reactions with O_2_, H_2_O, and other components of the material, including disproportionations of radical pairs) ensure that in most polymers, mechanochemical dissociation of an inert bond inevitably increases the fracture probability of the whole network [10].

Hydrogen, ionic, and many coordination bonds, as well as several types of covalent bonds, are dissociatively labile and are in dynamic equilibrium with their scissile products even in unstressed materials [27], although the equilibrium may lie far towards the bonded forms [28]. Since tensile load usually (but not always [29]) reduces the dissociative stability of a bond [30], this equilibrium shifts increasingly towards the dissociation products in the material under any mechanical load other than isotropic (hydrostatic) compression. Once the mechanical load disappears, these labile bonds reform spontaneously, albeit at a rate that may be limited by macromolecular reordering rather than the intrinsic kinetics of the bond formation.

Chemically crosslinked networks containing dissociatively labile bonds above a certain threshold fraction can fracture predominantly by dissociation of these labile bonds, leaving their inert counterparts intact [31,32]. This is true whether the labile bonds are located predominantly in the backbones of chain segments between crosslinks or at the crosslinks themselves. Regenerating these labile bonds appears sufficient to recover the bulk mechanical properties of the fractured sample, at least under favorable scenarios. Since such regeneration requires traversing low activation barriers, self-healing occurs spontaneously at practical temperatures (although chain diffusion may limit the rate). The price of this ease of self-healing is the low tensile strength (<1 MPa) of such polymers, which greatly limits their technological potential [33]. Self-healing and road-to-applications of such polymers have been extensively reviewed in the literature and we do not discuss them further here.

When dissociatively labile backbone bonds are present at below-the-threshold fractions, they act primarily as sacrificial bonds whose dissociations dissipate mechanical energy as heat, thereby increasing the material toughness. Sacrificial bonds modify the material response to loads well below the critical value, but their effect on the tensile strength or tearing energy is limited [28,34,35,36]. In the reported polymers, regenerating only the sacrificial bonds restores up to 80% of the energy-dissipating capacity of the intact sample below a threshold load [37]. Conversely, existing data suggest that recovery of all the other properties requires regenerating the original density of the inert load-bearing backbone bonds [12,14,38]. The autonomic approaches to do so reported to date rely on either the formation of new inert covalent crosslinks between the existing backbones (either intact or fractured) or on extending a fractured backbone by polymerization of monomer dissolved in the polymer.

Here, we review these reported chemical strategies for the autonomic regeneration of dissociatively inert backbone bonds. Compared to the well-established non-autonomic regeneration or spontaneous reformation of labile bonds (including dynamic covalent bonds), all of which have been exhaustively reviewed, few plausible approaches to autonomic regeneration of “strong” backbones have been described, almost all in the last decade. This contrast attests to the unique challenges that regenerating the strongest components of a molecular network presents for molecular design, chemical implementation, and physicochemical characterization of the resultant behavior. Our review is motivated by the topical nature of autonomic self-healing, the lack of a comprehensive analysis of the requisite strategies in the literature, the fundamental and conceptual insights that learning how to spontaneously regenerate dissociatively inert backbone bonds offer, and utilitarian considerations. The latter includes our assessment that autonomic synchronous regeneration of mechanically strong molecular networks offers the broadest potential for generating self-healing polymers of practical importance, as well as the potential technological value of strategies to increase the fatigue lifetimes, failure stresses, and tearing energies of polymers without affecting their mechanical behavior at loads far below critical, where most polymers are designed to operate routinely. We note that all approaches analyzed below are demonstrations of bond-forming chemistry, with varying degrees of potential to be exploited in yielding practical self-healing materials rather than mature solutions for translation into practical self-healing materials.

## 2. New Load-Bearing Bonds from Small-Molecule Dopants

**Figure 3:**
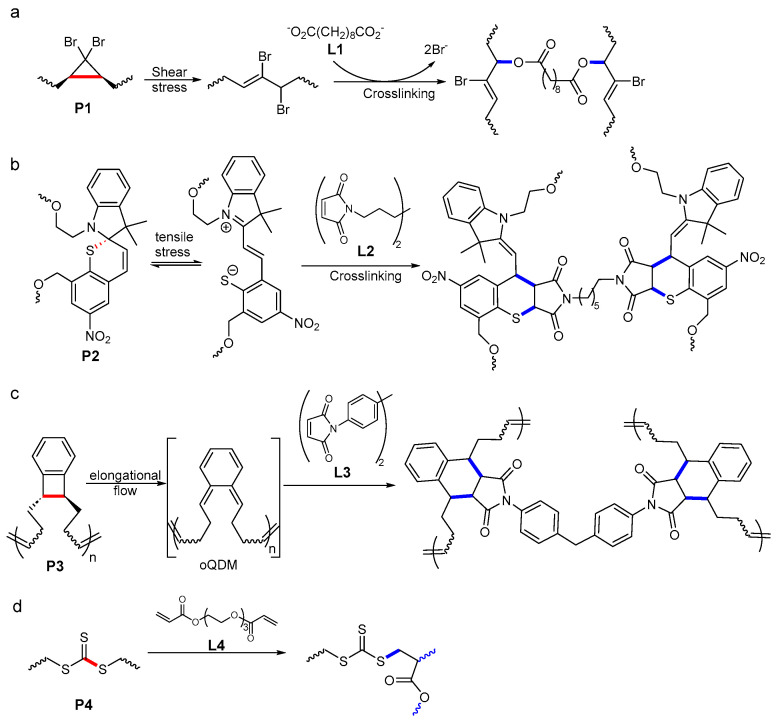
Reported approaches for regenerating load-bearing backbone bonds using small-molecule reagents. The scissile backbone bonds are in red, and the newly formed bonds are in blue. Crosslinking chemistries: (**a**) esterification of mechanochemically generated allylic bromides by small-molecule biscarboxylate solute; (**b**) formal thiol/ene addition of maleimide to mechanochemically generated thiomerocyanine; (**c**) spontaneous Diels-Alder addition to highly-reactive mechanochemically generated diene, oQDM; (**d**) radical acrylate polymerization initiated by mechanochemically generated thiyl macroradical.

We are aware of five reported implementations of this approach (Figure 3), two of which were demonstrated in bulk solids.

The shearing melt of P1 (polydibromocyclobutane) containing the dissolved ditetrabutylammonium salt of sebacic acid, L1, at 40 °C produced an insoluble material, with an elastic modulus ~20-fold higher than P1 [39]. Conversely, under identical conditions, shearing P1 without the solute reduced the average size of the chains, indicating that the shear load was sufficient to fracture chains mechanochemically. The increased modulus and reduced solubility of the material sheared in the presence of L1 were attributed to extensive crosslinking. This is thought to result from mechanochemical isomerization of dibromocyclobutanes to allylic bromides, which requires considerably lower force than the chain fracture [40] and is likely experienced by many chains that do not fracture, followed by esterification of the dicarboxylate additive by two different chains (Figure 3a). This esterification was confirmed by the appearance of the IR absorption at 1720 cm^−1^, corresponding to the ester functional group, with concomitant reduction in the intensity of the absorbance of carboxylate at 1560 cm^−1^. The capacity of the mixture of P1 and L1 to crosslink under mechanical load was further supported by the observation of a bifurcation of the mass distribution of P1 in sonicated solutions of P1 + L1 mixtures, with the appearance of both larger and smaller chains than those before sonicating [39].

Both the sheared and sonicated mixtures continued to crosslink after the removal of mechanical loading, indicating that the mechanochemical reaction, which produces allylic bromides, is considerably faster than their subsequent reactions with the biscarboxylate. This means that this crosslinking approach can overcome the destructive chain fracture only for specific, potentially narrow combinations of the concentration of the linker and the flux of mechanical energy. Since continuous crosslinking both depletes L1 and increases how efficiently external mechanical load is coupled to mechanochemical reaction rate [41], the loading scenarios in which this approach may be made practical depend strongly on the respective limits, which remain to be disclosed. Another potential complication in assessing how generalizable this approach arises from the unknown contribution of localized heating, which is inevitable in polymer melt extrusion, to the crosslinking kinetics.

In contrast, preliminary evidence suggests that under tensile load, the rate of crosslinking of spirothiopyran-modified polyurethane, P2, containing dissolved small-molecule bismaleimide, L2, is limited by the kinetics of the mechanochemical step rather than the subsequent thermal addition [42]. Under ambient conditions, spirothiopyran (SPT) exists in dynamic equilibrium with its thiomerocyanine isomer (TMC, Figure 3b) separated by a barrier of ~22 kcal/mol [43]. Tensile load shifts the equilibrium toward TMC, which is evident by the appearance of its distinct green color when L2-free P2 is stretched. Conversely, P2 doped with L2 (~0.1% by mass) bleached when stretched and acquired a new IR absorption at 1655 cm^−1^, which was attributed, based on model studies, to the thiol/ene adduct of maleimide and TMC. This rapid addition, accompanied by the bleaching of the yellow color of SPT, was confirmed by characterizing the composition of a sonicated solution of P2 + maleimide with UV-Vis absorption and NMR spectroscopies and GPC. Replacing maleimide with L2 caused the sonicated solution to gel rapidly, as expected [42].

The above example appears to be the only unambiguous implementation of this self-healing strategy, which is both autonomic and synchronous, in a bulk polymer solid.

Practically interesting crosslinking kinetics may also be achievable by replacing TMC with highly reactive *ortho*-quinodimethide (oQDM, Figure 3c) generated by mechanochemical isomerization of benzocyclobutadene, but this chemistry has only been demonstrated in sonicated solutions [44]. Sonicating semi-dilute solutions of P3 gradually increased the average size of the dissolved polymer, in contrast to the expected reduction in the chain length observed at lower polymer concentrations. The increase was attributed to the dimerization of oQDM moieties on different chains, but no information about chain topology was reported. The average size plateaued at ~3 times that of the intact polymer, potentially indicating a steady state in which the rate of mechanochemical chain fracture matched the rate of chain additions. Repeating sonication in the presence of bismaleimide, L3, caused rapid gelation, presumably by the DA addition of maleimide to oQDM. The low solubility of L3 may contribute to this rapid gelation, complicating inferences about which of the two sequential steps (mechanochemical isomerization vs. additions of unloaded reactants) is rate-determining [44].

A somewhat different approach to mechanochemical molecular network regeneration exploited the long-known observation that macroradicals generated by mechanochemical fracture of overstretched polymer backbones can initiate radical polymerization of suitably activated monomers, such as acrylates and styrenes [45]. A solution of either poly(n-butyl acrylate) or poly(n-butyl acrylate) containing a single dissociatively labile trithiocarbonate sacrificial backbone group, P4, and bisacrylate crosslinker L4 (Figure 3d) gelled upon sonication, presumably reflecting radical oligomerization of the acrylate initiated by a thiyl macroradical from mechanochemical fracture of the backbone C-S bond [46].

**Figure 4:**
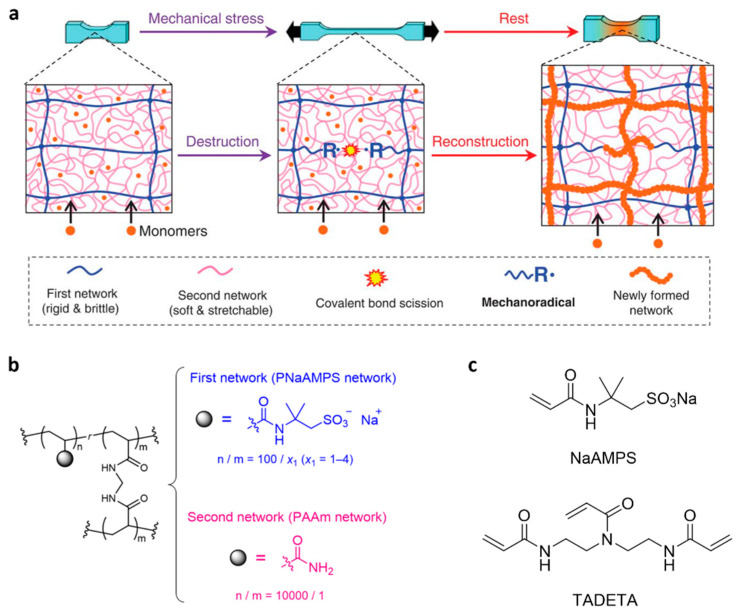
Molecular network generation by polymerization of acrylates initiated by mechanochemically generated macroradicals: (**a**) cartoon representation of the experimental design; (**b**,**c**) chemical structures of the polymers and polymerizing acrylate solutes. Adapted with permission from the authors of [47].

A more practical implementation of mechanochemically initiated radical polymerization was demonstrated in acrylate hydrogels (Figure 4) [47]. Under Ar, stretching a double-network (DN) acrylamide hydrogel containing molar concentrations of mono- and bisacrylates, followed by resting, nearly doubled the failure stress of the sample, and increased its elastic modulus > 10-fold. In contrast, the same DN hydrogel lacking the dissolved acrylates softened when similarly stretched, as expected. The strengthening was attributed to the conversion of the dissolved acrylates into a new brittle molecular network, thereby more than compensating for the loss of load-bearing segments of the original primary network that fractured during stretching. The macroradical initiators of this polymerization were thought to result exclusively from the primary network. The lack of a similar network regeneration in a single-network gel of identical composition was attributed to the insufficient concentration of macroradicals generated in it by stretching.

Among the examples discussed here, in situ polymerization (Figure 4) appears to be the only one to allow some control of both the kinetics and the resulting density of regenerated load-bearing bonds in a solid by adjusting the concentration of the monomers and the ratio of mono- to bisacrylates fed into the gel. However, it is also the least likely of all reported-to-date implementations of this strategy to yield practical self-healing materials. Regenerating the original mechanical properties required kinetic chain lengths (i.e., the number of monomers incorporated into the growing chain before it terminates) of 10^2^–10^3^, which has so far been only demonstrated in multinetwork hydrogels [47]. Only these materials seem to produce sufficient concentrations of macroradicals under tensile load and allow sufficient concentrations of dissolved monomers and the necessary fast diffusion. It is unclear to us how these constraints can be lifted. Simultaneously, the existing and prospective uses of weak hydrogels (tensile strength < 1 MPa) are not compatible with them containing highly mobile reactive (and hence toxic) additives [33], whereas meaningfully higher strengths require network densities that likely allow neither the sufficient concentration of the monomer solutes nor sufficiently fast diffusion [38].

## 3. New Load-Bearing Bonds from Sacrificial (Labile) Bonds

**Figure 5:**
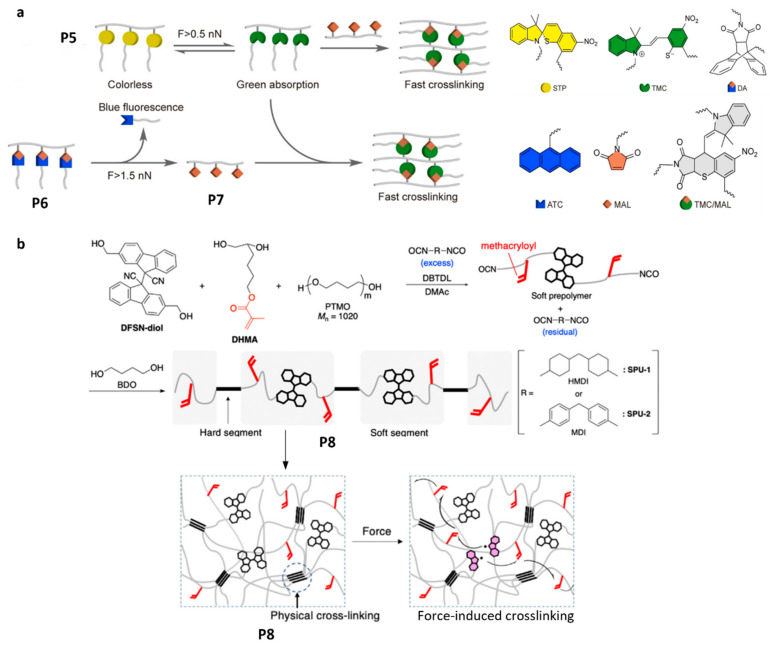
Polymer designs for load-induced crosslinking without dopants: (**a**) crosslinking by thiol/ene addition of mechanochemically activated comb polymers; (**b**) crosslinking by radical oligomerization of acrylate side chains. Adapted with permission from the authors of [43,51].

The most obvious limitation of the approach discussed in the preceding section is the need for large amounts of small-molecule reactants (bifunctional crosslinkers or monomers) dissolved in a polymer sample to endow it with self-healing capability. Recent work demonstrates implementations of the two chemistries discussed above (thiol/ene addition and radical acrylate polymerization) without the use of small-molecule solutes, albeit at the cost of more complex polymer topologies and compositions (Figure 5).

Sonication of dilute solutions of comb polymers P5 and P6 (Figure 5a) produced insoluble material, which was attributed to the formation of new load-bearing bonds among individual dissolved chains by a three-step reaction cascade [43]:

(a) Reversible mechanochemical isomerization of spyrothiopyran (STP, yellow in Figure 5a) to thiomerocyanine (TMC, green);
(b) Irreversible conversion of P6 into P7 by mechanochemical dissociation of multiple DA adducts (Figure 5a) per chain;
(c) Fast non-mechanochemical addition of the maleimides of P7 (orange) to TMC of P5.

The comb polymers P5 and P6 used in this work contained a short main backbone with multiple three-way branching points, each connecting to a longer side chain. The use of a comb polymer as the precursor of maleimide-bearing macromolecule P7 offers multiple advantages. First, it is a clever work-around for the major challenge of relying on mechanochemical chain fracture to generate reagent(s) for downstream reactions [48] (or to deliver small-molecule payloads [49]). Because the probability of a backbone becoming overstretched and reacting mechanochemically scales as close to a cube of the chain contour length [50], incorporating even multiple scissile moieties into a backbone of a linear chain rarely allows activation of more than three such moieties over practical loading timescales. In a comb polymer, every pair of side chains has an approximately equal probability of becoming the overstretched backbone. A single backbone fracture (which in P5 occurred primarily by dissociation of the DA adduct due to its positioning close to the center of the overstretched backbone) reduced the probability of the remaining comb polymer (now having one fewer side arm) by a few percent rather than by ~2^3^-fold of the linear counterpart. Thus, a comb polymer allows the activation of multiple scissile mechanosensitive moieties per chain at a rate that decreases only weakly with the fraction of the remaining such moieties. Because STP is a non-scissile mechanoresponsive moiety, the use of the comb topology for its activation (P5) was unnecessary and reflected synthetic convenience [43].

The second advantage of this approach is the ability to “gate” the kinetics of the non-mechanochemical crosslinking reaction by mechanical load [36], which avoids both wasting crosslinking agents outside the overstressed volumes of the loaded material and deleterious embrittlement of the sample. Whereas the DA adduct is dissociatively inert on practical timescales at single-chain forces of <2.3 nN (vs. ~4.3 nN for aliphatic (H_2_)C-C(H_2_) bond fracture), the threshold force of STP isomerization is ~1.2 nN [43]. This combination has two consequences. In volumes of material at high risk of fracture and thus at high need for network regeneration (or reinforcement), i.e., that experience average single-chain forces of >2.5 nN, the high concentration of TMC ensures rapid crosslinking. Simultaneously, in less-stressed volumes, the maleimide generation is too slow to support crosslinking regardless of the concentration of reactive TMC moieties. Because STP/TMC isomerization is reversible, any TMC that was generated but not consumed in crosslinking reverts to STP as soon as the load dissipates and is thus available for crosslinking. Since the threshold force of the DA adduct dissociation is tunable by peripheral substitution of the anthracene moiety, the average mechanical stress triggering the crosslinking is adjustable. Thus, although these DA adducts serve as sacrificial bonds because they dissociate at a considerably lower force than the load-bearing bonds do, it is sufficiently mechanochemically inert to affect material behavior only at close-to-critical stresses. This attribute contrasts with the approaches discussed in the preceding section that allow very limited control of crosslinking kinetics, which both wastes the limited amount of the bifunctional linker and leads to undesired embrittlement of the polymer even after the load dissipates.

**Figure 6:**
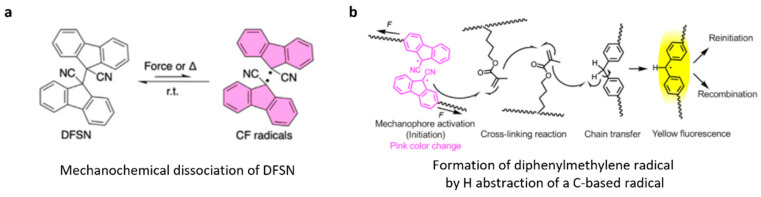
Chemistry enabling load-induced crosslinking of polymers, combining the sacrificial moiety DFSN in the backbone and acrylate side chains. (**a**) DFSN dissociation; (**b**) evidence of a radical chain in compressed material from transient generation of a fluorophore. Adapted with permission from the authors of [51].

Similarly, upon uniaxial compression, segmented polyurethanes containing multiple difluorenylsuccinonitrile moieties per backbone and methacryloyl groups as side chains (P8, Figure 5b) increased their storage module and became insoluble [51]. The effect was attributed to crosslinking by radical oligomerization of pendant acrylates initiated by cyanofluorene radicals from mechanochemical backbone fractures by dissociation of DFSN moieties (Figure 6). Radical diffusion across the molecular network was confirmed using a related polymer bearing H-atom donor sites (diphenylmethylene). EPR spectroscopy revealed the presence of both cyanofluorene and diphenylmethylene radicals in compressed samples, the former resulting from mechanochemical dissociation of DFSN (Figure 6a) and the latter from H-atom abstraction by a C-based radical derived from radically-initiated acrylate oligomerization (Figure 6b) [51] (the likelihood of H-atom transfer from diphenylmethylene to cyanofluorene was ruled out by DFT calculations).

The extensive mechanically induced crosslinking of polymers containing a high fraction of dissociatively labile backbones (thanks to the incorporation of DFSN moieties) is remarkable since DFSN is both kinetically and thermodynamically unstable with respect to its dissociation products at forces < 1 nN [28]. The capacity to obtain the necessary kinetic chain lengths probably reflects the high concentration of the pendant acrylates in these materials and the high 1:4 molar ratio of the initiator precursor to acrylate. Unfortunately, the lack of data on the kinetics of acrylate oligomerization in these materials, as well as the resulting crosslinking densities and their dependence on the concentrations of acrylates and DFSN/acrylate ratios, preclude further analysis of the potential of this implementation to yield practically interesting autonomic self-healing solutions.

## 4. New-Load-Bearing Bonds by Direct Chain-to-Chain Addition

Integrating the reactive moieties that generate new load-bearing bonds into polymers, instead of adding them as dopants, offers obvious advantages for practical applications. The implementations discussed in the preceding section, however, come at a cost of polymer compositions and/or microstructures that are impractically complex, making the polymers prohibitively expensive and difficult to process, and reducing the mechanical stability of the molecular network due to the need to replace a large fraction of load-bearing bonds by sacrificial bonds.

**Figure 7:**
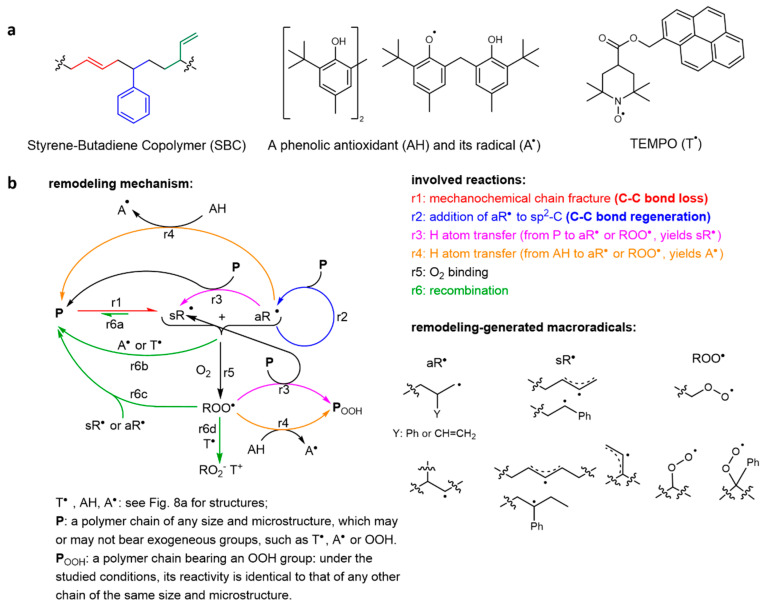
A random styrene/butadiene copolymer spontaneously forms new load-bearing backbone bonds under destructive shear loads. (**a**) The structure of the copolymer and the dopants whose effect on the bond-forming kinetics was quantified. (**b**) The molecular mechanism sufficient to account for the behavior of the material under diverse loading conditions. Adapted from [26] with permission.

These reasons explain the critical importance of the recent demonstration of spontaneous regeneration of load-bearing bonds in compositionally simple styrene/butadiene copolymers (SBC, Figure 7a) as well as the detailed quantitation of the underlying kinetics and thermodynamics, and plausible means to control them. The simple composition and microstructure, which are often perceived as primitive or lacking innovation by academic researchers, are a major advantage for commercializing these findings because SBCs are widely used and highly efficient industrial-scale processes for synthesis and processing of such copolymers are well established [26].

**Figure 8:**
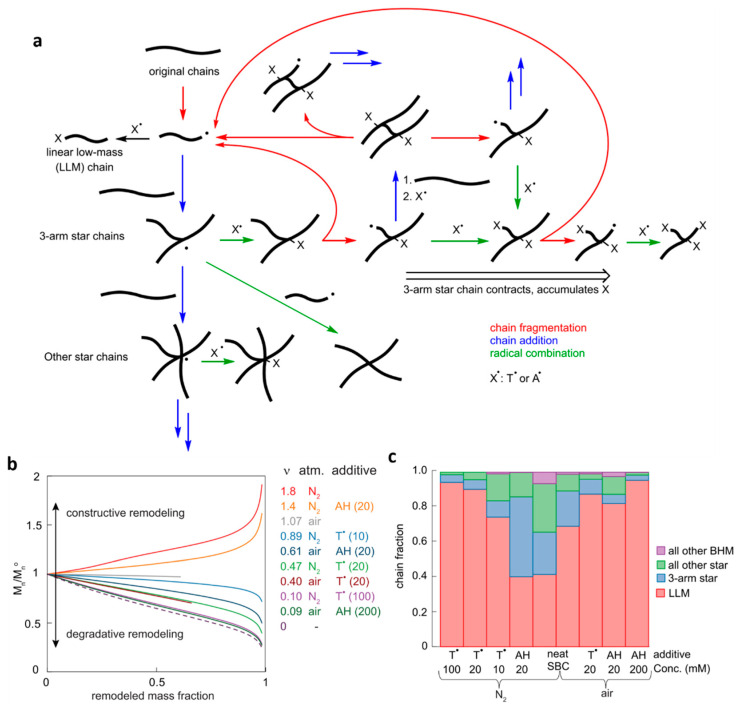
Key attributes of autonomic synchronous self-healing in sheared styrene/butadiene copolymers. (**a**) the competition between chain fracture and chain addition and the resultant accretion of multiple small-molecule radical scavengers in accumulating branched chains. (**b**) The change in the average size of the polymer chain for melts containing different small-molecule solutes. (**c**) The steady-state distribution of chain topologies in sheared melts (LLM = linear low-mass chains; BHM = branched, high-mass chains). Adapted from [26] with permission.

In this initial demonstration of autonomic synchronous regeneration of load-bearing bonds in compositionally simple polymers, melts of low-dispersity linear SBC were sheared at 10 °C in both N_2_ atmosphere and air, including in the presence of small-molecule dopants that would normally be present in commercial formulations (e.g., a phenolic antioxidant, Figure 7a). Careful extensive kinetics studies yielded a detailed, predictive mechanism of the new bond formation (Figure 7b) and quantitative estimates of the efficiency of this regeneration under diverse practically relevant scenarios (Figure 8). Under all conditions studied, the mass distribution of the sheared polymer bifurcated, with chains that were shorter than the starting material suggesting backbone fracture, and the (much) longer, including highly branched, chains, indicating the generation of new backbone bonds. The larger chains were attributed to terminal macroradicals from mechanochemical chain fracture (Figure 8a) growing by sequentially adding adjacent chains through radical addition to sp^2^-C atoms.

Previously, unactivated C=C bonds, particularly backbone C=C bonds, were thought to be of limited value for backbone bond regeneration under ambient conditions due to their low reactivity towards C-based radicals (e.g., uncatalyzed radical polymerization of C_2_H_4_ or propylene requires high temperatures and/or pressures and most unsaturated polyolefins do not crosslink on practical timescales at temperatures < 60 °C even in the presence of organic peroxides as chain initiators). Consistent with this expectation, in sheared SBC only, alkyl macroradicals (aR^•^ in Figure 7b) initiated the bond-forming reaction cascades. Mechanochemical fracture of an SBC chain yields a mixture of alkyl and stabilized (allylic and benzylic, sR^•^) radicals, whose ratios depend on the fracturing force, the ratio of 1,4- and 1,2-enchained butadienes (red and green moieties in Figure 7a) and styrene, and the presence of any small-molecule radical scavenging dopants. The capacity of unactivated C=C bonds of SBC to support constructive network remodeling under mechanical load illustrates the broader points that crosslinking existing chains is a more efficient means of enhancing the mechanical stability of a network than forming new backbones by monomer polymerization or chain interdiffusion.

This work is the only example where the relative contributions of destructive and constructive remodeling of a mechanically loaded material were quantified, as the average number of new load-bearing backbone bonds formed per each mechanochemically fractured one, ν (Figure 8b). Below-unity values of ν signify the net loss of load-bearing bonds, i.e., material degradation, whereas ν > 1 corresponds to self-strengthening. Despite larger branched chains being formed under all studied conditions, the measured values of ν varied from 1.8 (effective self-strengthening) in neat SBC sheared under N_2_ to ~0.1 (effective degradation) for SBC saturated with small-molecule radical scavengers (Figure 8b). This illustrates that the appearance of apparently growing chains alone does not rule out extensive mechanical degradation. Conversely, the steep scaling of the chain fracture probability with the effective contour length and degree of branching of a chain limits ν to <2 because the largest chains fracture preferentially, thereby avoiding damaging embrittlement of the self-healing material.

Another oft-cited perceived drawback of relying on radical-mediated chemistry to regenerate dissociatively inert (load-bearing) bonds is the susceptibility of many such reactions to aerobic inhibition. For example, the double-network acrylate-saturated hydrogels discussed in Section 2 self-heal only under Ar, which further limits their potential applications. SBC sheared in air manifested lower ν (1.1) than under N_2_ (1.8) but still comfortably higher than is needed to maintain a constant density of load-bearing bonds (ν = 1). In aerobically sheared SBC, stabilized macroradicals, which do not initiate the bond-forming cascades, protect the bond-forming alkyl macroradicals from O_2_ by two mechanisms. First, they bind O_2_ to form peroxyl radicals. Second, these peroxyl radicals abstract H-atoms from adjacent chains, generating a mixture of alkyl and stabilized internal macroradicals. Thus, stabilized macroradicals both reduce the steady-state concentration of O_2_ and increase the steady-state concentration of alkyl macroradicals that initiate the bond-forming cascades. Conversely, under N_2_ these stabilized radicals enable fast migration of unpaired electrons along backbones and between backbones by H-atom hopping, which leads to some radical reactions outside the overstressed volume of the material.

Importantly, the number of newly formed backbone bonds per fractured bond was independent of the degree of remodeling for all conditions that produced constructive remodeling, which was attributed to the newly formed C-C bonds being chemically indistinguishable from the fractured bonds.

Detailed microkinetic simulations using mechanochemical kinetics of key steps derived from DFT calculations and validated against the measured product distributions suggested that only three distinct regimes of mechanochemical remodeling of a loaded polymer may exist, depending on the number of newly formed backbone bonds per fractured bond. Although the conclusions were derived explicitly for non-crosslinked polymers, they are likely valid for any chemistry that has so far been identified as enabling autonomic regeneration of mechanically lost load-bearing bonds. A conventional polymer that was not designed for self-healing, such as polystyrene, may reach ν ~ 0.3 due to a fraction of mechanochemically generated macroradicals from backbone fractures recombining into a chain of approximately original sizes, in competition with other irreversible reactions that do not yield new backbone bonds.

For polymers whose composition supports chain-to-chain additions yielding branched chains (e.g., unsaturated polyolefins, P1–P3, Figure 3, P5 + P6 and P7, Figure 5), this addition is too slow to compensate for the increased susceptibility of such chains to mechanochemical fracture (Figure 8) up to ν < 0.6. As a result, the mass fraction of chains containing newly formed backbone bonds remains <5%, and they affect little the bulk mechanical properties of the remodeling polymer. At 0.6 < ν < 1, branched chains accumulate until their mass fraction exceeds a certain threshold (which depends on loading scenarios), after which this fraction (and the weight-average chain mass) starts gradually decreasing while the dispersity of chain sizes and branching degrees increases. Such non-monotonic changes are sometimes observed during the reactive processing of polymer melts. Finally, at ν > 1, the average chain size, branching ratio, and sample dispersity increase as the initial chains are converted (primarily) to larger branched chains. This regime is stable for polymers such as SBC in which bond forming does not require a constant supply of reactive moieties (whether as dopants or incorporated into polymer chains) but is estimated to be transient for the other chemistries discussed above and switch to degradation once the reactive moieties are depleted. In sheared SBC, the three predicted regimes were tested experimentally by changing the concentration of dopants to access each regime.

## 5. Summary and Outstanding Challenges

We define autonomic self-healing as the spontaneous regeneration of mechanochemically fractured load-bearing backbone bonds without any additional free energy input. When the density of the newly formed load-bearing bonds exceeds that in the pristine polymer, self-strengthening may result, whereby the elastic modulus, fracture stress, and/or tearing energy of the loaded material increases with accumulated load. We further distinguish synchronous self-healing, which counteracts mechanical degradation in real time in the material that remains in productive use and continues to sustain useful loads; and asynchronous self-healing, which occurs only in an unloaded partially degraded material, which requires removing it from active use for a dedicated “regeneration” phase. To date, autonomic self-healing remains to be achieved in an elastomer of practical interest.

Nonetheless, the reported findings allow broad generalization of the criteria that an approach to an autonomic synchronous regeneration of load-bearing bonds needs to meet to be of practical interest. Aside from the considerations of cost, synthetic accessibility, processibility, and specific performance characteristics that apply to any material for potential commercialization, practically relevant self-healing likely requires the following:

(a) A functionally inexhaustible (or constantly regenerating) supply of precursors for the bond-forming reactions.
(b) Self-regulating crosslinking kinetics that allow the rate of new backbone bond formation to match the rate of bond fracture and to avoid embrittlement.
(c) Means of tuning the (initial or steady-state) load-induced crosslinking kinetics without the need to modify the chain composition, topology, and microstructure for each load profile.
(d) Engineering self-healing capacity without reducing the mechanical stability of the molecular network.

Direct radical chain-to-chain addition in unsaturated polyolefins comes closest to satisfying these conditions. For example, in stressed unsaturated polyolefins, the new load-bearing bonds form by the addition of a mechanochemically generated macroradical to an sp^2^-C of a C=C bond in an adjacent chain. In a material that experiences sufficient load to degrade, the regeneration of fractured backbones does not meaningfully deplete either reactant. The concentration of C=C bonds in such materials is 1–10 M, whereas the depercolation concentration (the minimum number of network bonds that need to fracture for this volume of material to develop a crack) is on the order of 1 μM [10]. The reaction yields new C-C bonds that are chemically indistinguishable from the fractured bonds and, therefore, are as likely as the original bonds to yield a macroradical competent to initiate another C-C bond generation. This attribute also makes self-healing in unsaturated polyolefins self-regulating by ensuring that the fracture susceptibility of the original and the newly formed chains follow the identical dependence on chain size and branching ratio. Unsaturated polyolefins likely meet the third criterion because the efficiency of self-healing, which we define as the number of backbone bonds formed per each mechanically fractured bond, depends predictably on the concentration of a commercial antioxidant that is already included in typical polymer formulations. Finally, self-healing is intrinsic to these polymers because it does not rely on network-weakening sacrificial backbone bonds.

All other chemistries reported to date for autonomic self-healing fail one or more of the above criteria, with all relying on a limited supply of precursors for the new bond formation and all but one allowing no control over the crosslinking kinetics in bulk. Although several chemistries discussed above make the material either mechanochromic [42,43,51] or capable of autonomic growth [47], which enabled interesting exploitations, these properties are, at best, tangential to self-healing.

A broader conclusion from the analysis of the existing approaches to creating self-healing materials is that sophisticated (and difficult-to-synthesize) monomers, complex chain topologies, and microstructures do not necessarily produce improved functionality. Relatively simple compositions and chain topologies can yield very rich behavior under mechanical load that remains poorly understood and, if properly studied, could constitute a more plausible means of creating practical, exploitable, self-healing polymers.

The dearth of mechanistic kinetic studies of mechanochemical remodeling of stressed polymers, even the simplest one, attests to the challenges of characterizing such remodeling at the molecular scale but also to a historical lack of experimental and computational techniques and experimental designs to support mechanistic inferences. The situation is improving with two distinct developments. First, the SBC study [26] described a new quantitative framework to support mechanistic investigations of remodeling polymers, which is generalizable to any polymer whose composition, topology, and microstructure can be characterized in sufficient detail. The detailed molecular insights into the self-healing of unsaturated polyolefin it produced illustrate this framework’s value. It relies on the systematic and internally consistent reduction in the intractable complexity of a remodeling polymer, with its millions of structurally unique components, to a small subset of reactions primarily responsible for the remodeling. From this subset, suitably complex reaction networks can be built systematically and automatically without sacrificing chemical information or atomistic detail inherent in alternative approaches, e.g., lumped kinetic models. Second, increasingly sophisticated attempts to apply the tools of physical organic chemistry to relate load-induced changes in bulk material properties to the load-dependent kinetic and thermodynamic stabilities of the constituent monomers [12,13,14,22,31,32,38,52,53] have yielded provocative ideas of how bulk mechanical load distributes across the stressed molecular network and how molecular events contribute to chain propagation or sample fracture.

These developments increase our confidence that the largely qualitative observations that currently dominate the study of polymer mechanochemistry in melts and solids will be subsumed by detailed quantitation of product distributions, reaction kinetics, and structure/reactivity relationships. These are essential to moving from the empirically driven and exploratory design of autonomic self-healing polymers that rely on custom monomers, complex compositions, chain topologies, and microstructures to the systematic exploration of strategies to endow existing engineering and specialty polymers with autonomic self-healing properties.

## Data Availability

All data discussed in this review are taken from the referenced literature.
